# Spatio-Temporal Variations of Satellite-Based PM_2.5_ Concentrations and Its Determinants in Xinjiang, Northwest of China

**DOI:** 10.3390/ijerph17062157

**Published:** 2020-03-24

**Authors:** Wei Wang, Alim Samat, Jilili Abuduwaili, Yongxiao Ge

**Affiliations:** 1 State Key Laboratory of Desert and Oasis Ecology, Xinjiang Institute of Ecology and Geography, Chinese Academy of Sciences, Urumqi 830011, China; wangwei177@mails.ucas.ac.cn (W.W.); alim_smt@ms.xjb.ac.cn (A.S.); geyx@ms.xjb.ac.cn (Y.G.); 2 Research Center for Ecology and Environment of Central Asia, Chinese Academy of Sciences, Urumqi 830011, China; 3 University of Chinese Academy of Sciences, Beijing 100049, China

**Keywords:** PM_2.5_, spatio-temporal, geographical detector method, Xinjiang

## Abstract

With the aggravation of air pollution in recent years, a great deal of research on haze episodes is mainly concentrated on the east-central China. However, fine particulate matter (PM_2.5_) pollution in northwest China has rarely been discussed. To fill this gap, based on the standard deviational ellipse analysis and spatial autocorrelation statistics method, we explored the spatio-temporal variation and aggregation characteristics of PM_2.5_ concentrations in Xinjiang from 2001 to 2016. The result showed that annual average PM_2.5_ concentration was high both in the north slope of Tianshan Mountain and the western Tarim Basin. Furthermore, PM_2.5_ concentrations on the northern slope of the Tianshan Mountain increased significantly, while showing an obviously decrease in the western Tarim Basin during the period of 2001–2016. Based on the result of the geographical detector method (GDM), population density was the most dominant factor of the spatial distribution of PM_2.5_ concentrations (*q* = 0.550), followed by road network density (*q* = 0.423) and GDP density (*q* = 0.413). During the study period (2001–2016), the driving force of population density on the distribution of PM_2.5_ concentrations showed a gradual downward trend. However, other determinants, like DEM (Digital elevation model), NSL (Nighttime stable light), LCT (Land cover type), and NDVI (Normalized Difference Vegetation Index), show significant increased trends. Therefore, further effort is required to reveal the role of landform and vegetation in the spatio-temporal variations of PM_2.5_ concentrations. Moreover, the local government should take effective measures to control urban sprawl while accelerating economic development.

## 1. Introduction

The atmospheric particulate matter with a diameter of 2.5 μm or less (PM_2.5_) is the common indicator of air quality. Most of PM_2.5_ is emitted from power plants, industries, automobiles construction sites, wild fires, and so on. Due to its multitudinous emission sources, PM_2.5_ pollution has gradually become a worldwide environmental problem. PM_2.5_ contains some compounds, such as polycyclic aromatic hydrocarbons (PAHs), heavy metals, and microorganisms [1]. Meanwhile, because of its tiny size, PM_2.5_ has a long residence time in the atmosphere and can penetrate to the lower airways [2,3]. According to World Health Statistics 2016, the World Health Organization (WHO) estimated that more than 90% people living in cities breathed air containing high levels of PM_2.5_ in 2014 [4]. One of the most obvious problems induced by high levels of PM_2.5_ exposure is premature death [5]. Numerous epidemiological studies have shown the harmful effects of long-term PM_2.5_ exposure on human health [1,2,6,7,8,9]. Pun et al. [6] found that long-term PM_2.5_ exposure has a strong relationship with increased mortality from respiratory disease, lung cancer, and cardiovascular disease in US elderly. Li and Gao [2] found that particle pollution is significantly related to lung cancer mortality in China. Hoek et al. [7] demonstrated that PM_2.5_ pollution has been highly correlated with a few adverse health effects, such as respiratory and cardiovascular diseases. According to the Global Burden of Disease (GBD) in 2015, it is generally accepted that the air pollution caused by PM_2.5_ is the leading cause of non-communicable diseases [10]. With the rapid industrialization and urbanization process over the past three decades, atmospheric pollution caused by large energy consumption has become one of the most ubiquitous and concerning issues in China [11]. The air quality in more than three-quarters of cities in China exceeded the national air quality standards in 2016 [12]. In order to solve the air pollution problem, the Chinese government proposed a range of moves to control the air pollution, such as phase-out of backward production facilities, increasing urban green space, and so on. Although these moves have gotten some achievements, there also exist many problems [13].

In 2013, the Chinese government proposed “Action Plan on the Prevention and Control of Air Pollution” to aggressively control PM_2.5_ emissions from human activities. Meanwhile, the Chinese National Environmental Monitoring Center (CNEMC) started to establish a nationwide air quality monitoring network in China until the end of 2013. However, relatively sparse distribution of air quality monitoring sites and lack of long-term observation data have brought challenges to the study of the spatio-temporal variations of PM_2.5_ concentrations, especially in northwest China. Moreover, due to most of the sites located in densely populated cities, PM_2.5_ data with spatial continuity obtained by interpolation based on monitoring data easily have a “bull’s-eye” effect (higher values near observed location). Additionally, the ground monitoring data are too little to describe the spatio-temporal variation of PM_2.5_ concentrations. With the development of remote sensing techniques, PM_2.5_ concentration data based on remote sensing techniques have become increasingly popular in recent years [14,15,16]. By using the developed high-precision PM_2.5_ retrieved algorithms and statistical methods, global estimates of PM_2.5_ concentrations were calculated based on multi-source remote sensing data and ground-based sun photometer (AERONET) observations [5,17].

For the past several years, researchers have conducted numerous studies on the spatio-temporal variations of PM_2.5_ concentration and the influence of human activities and natural conditions on it [12,18,19,20]. For instance, Chu and Bilal [16] used integrated geographically temporally weighted regression (GTWR) and random sample consensus (RANSAC) models for mapping PM_2.5_ based on satellite-derived Aerosol Optical Depth (AOD) data in Taiwan. Wei et al. [20] investigated the relationships between PM_2.5_ and other air pollutants (SO_2_, NO_2_, PM_10_, CO, and O_3_) in Heilongjiang province based on geographically and temporally weighted regression (GTWR) models. Luo et al. [18] used the method of geographically weighted regression (GWR) to analyze the natural geographical and socio-economic factors of PM 2.5 concentrations in 343 cities across Mainland China. Lu et al. [21] employed the grey system correlation analysis method to analysis the main influencing factors of PM_2.5_ concentrations in China. Ji et al. [13] analyzed the correlation between satellite-based nighttime stable light (NSL) data and statistical PM_2.5_ emissions at the provincial level in China from 1992 to 2012. Xu et al. [22] investigated the response of the PM_2.5_ concentration to meteorological, underlying surface and socio-economic conditions in the Yangtze River Delta by Spearman correlation analysis, multivariate analysis of variance (MANOVA), and lasso regression. 

However, few studies could quantify the contribution of natural driving factors and socio-economic driving factors to PM_2.5_ concentrations from the spatial heterogeneity perspective. Furthermore, because the formation of PM_2.5_ pollution is a very complex process, there exist the interaction effects among multiple driving factors [21]. Thus, it is of interest to find a quantitative model or method that describes this spatio-temporal relationship between PM_2.5_ concentrations and its driving factors. A method based on spatial heterogeneity named the geographical detector method (GDM) may be a preferable method for exploring the factors influencing the spatio-temporal distribution of PM_2.5_ concentrations. GDM is a new statistical method to analyze the driving factors controlling the spatial patterns of various geographical phenomena [23]. According to the literature, GDM can not only quantitatively determine the relative importance of each driving factor both in spatial and temporal variation, but also address the joint effects of these factors on the spatio-temporal variation of PM_2.5_ concentrations. Therefore, GDM has attracted wide attention of the application from various fields with proven advantages [24,25,26].

Growing research in China has dedicated enormous effort focused on PM_2.5_ concentrations and its driving factors in developed regions, such as Beijing, Tianjin, Hebei, Nanjing, Shanghai [27,28,29]. Most of these regions are located in east-central China which has a higher population density and economic growth. However, with the awareness campaigns of environment protection over recent years, the central and eastern regions of China are paying more and more attention to the prevention and control of air pollution. Meanwhile, due to huge differences in the natural environment and socio-economic conditions between east and west regions, some pollution emitting industries located in eastern regions were moved to the west regions where there are more liberal environmental policies [14]. While promoting regional economic development and increasing employment, this kind of transfer changed the regional industrial structure and increased the enterprise pollution emissions. On the other hand, urban population density and the urban agglomeration scale experienced a rapid growth period [24]. Additionally, from the prospect of natural factors, frequent sandstorm activity and the long winter heating period significantly aggravate the air pollution in Xinjiang.

According to the first quarter 2016 air quality readings downloaded from the website of the China National Environmental Monitoring Center (CNEMC), 7 of the 20 worst cities in the country come from Xinjiang: Kashgar, Wujiaqu, Urumqi, Hetian, Kizilsu, Shihezi, and Aksu. In a recent Greenspace survey, government enacted many pollution limiting guidelines in the eastern China, but not in west. This regional policy difference had an unintended effect of encouraging polluting enterprises to move their investments to western provinces, such as Xinjiang. In the northern part of the Tianshan Mountain and the western margin of the Tarim Basin, about 10 million people have suffered from serious air pollution in the past decade [30]. Meanwhile, the North Tianshan Mountain Economic Zone (NTMEZ), the largest and the most comprehensive economic belt of China, is located in this region [31]. Xinjiang is also the core area of the ‘Belt and Road Initiative’. However, few studies explored the spatio-temporal variations of PM_2.5_ concentrations and its driving factors in northwest China, especially Xinjiang. Existing research mainly focuses on chemical characteristics analysis and source apportionment of PM_2.5_ pollutants in individual cities of Xinjiang. For example, Chen et al. [32] investigated the sources of heavy metals (HMs) and per fluorinated compounds (PFCs) in PM_2.5_ in Urumqi and Shihezi, two of the major industrial cities in northern Xinjiang. Turap et al. [30] measured the major components of ambient PM_2.5_ in four seasons in Dushanzi, finding that the mixing of anthropogenic aerosol sources and dust were the main sources of PM_2.5_.

Unfortunately, to the best of our knowledge, almost no research has been done to analyze PM_2.5_ spatial distribution and its driving factors from a regional perspective in Xinjiang [32,33,34]. Xinjiang is the largest administrative region as well as the largest arid land in China, where there are less precipitation and vegetation distributions. More than 26% of the land is covered by deserts where the main sources of dust storms are located. Recent studies have indicated that satellite-based PM_2.5_ concentration data can be used to understand the spatio-temporal variability of atmospheric pollutants in arid region [35,36,37,38,39]. For example, Munir et al. [39] used satellite-derived PM_2.5_ concentration data to analyze the spatial and temporal variability of PM_2.5_ in Saudi Arabia, finding that remote sensing can help better understand the spatial variability of atmospheric pollutants, especially on a large scale. In Xinjiang, the harsh environment and increasing city size are more likely to cause or enhance the accumulation of atmospheric pollutants. Meanwhile, most of people in Xinjiang lived in the oasis, which is a specific landscape in arid land. Although oasis accounts for 4–5% of the total area in Xinjiang, more than 90% of cultivated land, population, and 95% of GDP are concentrated within the oasis [40]. Therefore, oasis-intensive industrial activities and urban agglomerations are also the reasons for the rapid increase in air PM_2.5_ concentrations in the past decade. On the other hand, the increasing number of automobiles is one factor that should not be neglected. According to the China Statistical Yearbook 2019, private car ownership in Xinjiang was more than 3.29 million by the end of 2018, especially concentrated in big cities like Urumqi [41]. Accordingly, the spatio-temporal variation of PM_2.5_ concentrations is worth studying in Xinjiang. Meanwhile, identifying the natural and socio-economic determinants of PM_2.5_ concentrations will contribute to effectively solving air pollution problems in this region.

Therefore, based on global annual average surface PM_2.5_ concentration data in the long-time series (2001–2016), the purposes of this study were (1) exploring the spatio-temporal characteristics of PM_2.5_ concentrations by spatial autocorrelation analysis; (2) identifying the dominant factors responsible for spatio-temporal variations, especially the socio-economic factors; (3) quantitatively analyzing the interannual variations of the dominant power of PM_2.5_ driving factors. The main conclusions could be beneficial for developing environmental policy and constituting a regional development plan.

## 2. Materials and Methods 

### 2.1. Study Area 

**Figure 1:**
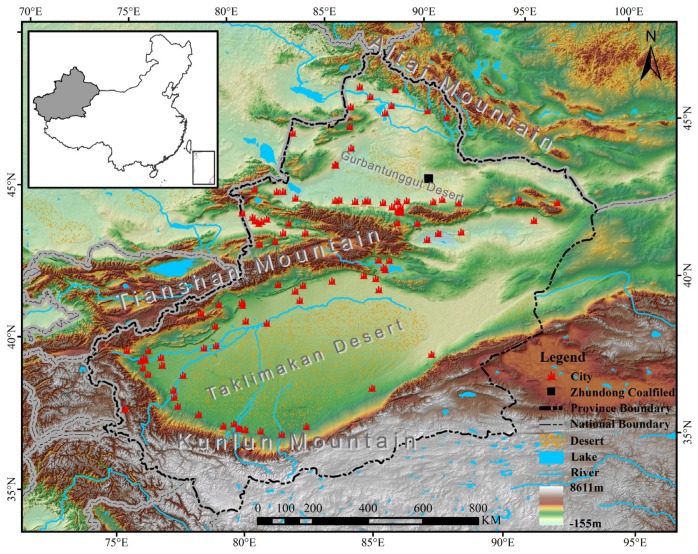
Study area.

Xinjiang (34°22′–49°33′ N, 73°22′–96°21′ E) which is located in northwestern China is the largest province in this country (Figure 1). Situated at the hinterland of the Eurasia continent, the total land area of Xinjiang is approximately 1.66 × 10^6^ km^2^, accounting for about 1/6 of China. Due to the influence of continental climate, the annual precipitation in Xinjiang is about 145 mm [42]. In addition, most of the effective precipitation is mainly concentrated in winter. Divided by three high-elevation mountain areas (Altai Mountain, Tianshan Mountain, Kunlun Mountain), Xinjiang has formed a unique mountain–oasis–desert landscape ecosystem (Figure 1). Affected by the spatial allocation of water resources, most cities in Xinjiang are surrounded by two deserts (Gurbantunggut Desert and Taklimakan Desert). Thus, the urban agglomerations in this area are mostly distributed by strips or rings. Although cities and urban agglomeration are playing key roles in regional economic development, various environmental problems begin to emerge under the influence of their intense human activities and fragile ecological environment. The air pollution caused by mixtures of industrial emission, vehicle emission, and fine dust particles has become an urgent problem in this region [30].

### 2.2. Data Source

**Figure 2:**
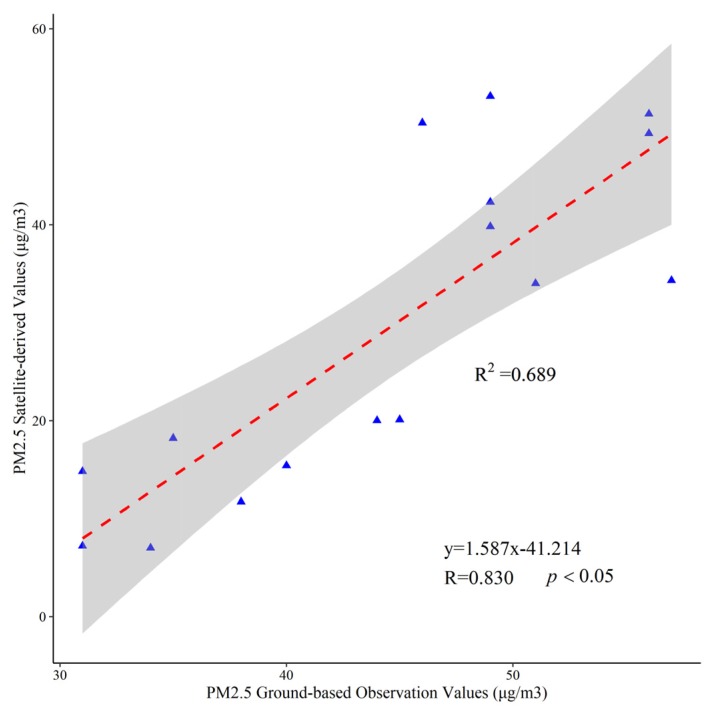
Linear correlation between PM_2.5_ ground-based observation values and satellite-derived values.

The global annual average surface PM_2.5_ concentrations grids, which is estimated by Aerosol Optical Depth (AOD) retrievals from multiple satellite products (MISR, MODIS-DT, MODIS-DB, MODIS-MAIAC, and SeaWiFS-DB), was used in this study [5]. The satellite-based gridded PM_2.5_ dataset has a spatial resolution of 0.01 x 0.01 degree, and it was combined with simulation (GEOS-Chem model) and calibration (geographically weighted regression) based on the ground photometer (AERONET) observations from 1998 to 2016. This PM_2.5_ concentration dataset, which is provided by the Atmospheric Composition Analysis Group (ACAG) at Dalhousie University, has been used in numerous studies at the regional and national scale [18,21,39,43]. Although the accuracy of the global annual average surface PM_2.5_ concentrations grid data has been validated by using the 210 global mean ground-level PM_2.5_ measurements collected from the literature, few measurements are located in the arid region, especially in Xinjiang. Considering the unique spatial heterogeneity of PM_2.5_, it was still necessary to evaluate the reliability of the dataset in this study area. In order to ensure continuity and integrity of PM_2.5_ concentration data, we collected the hourly official PM_2.5_ site monitoring data in Xinjiang provided by the China National Environmental Monitoring Center (CNEMC) from Jan 1st, 2015. We calculated the annual average PM_2.5_ ground-based observations based on 8 sites which are mainly concentrated in north Xinjiang (Urumqi, Changji, Bole, Yili, Wujiaqu, Tacheng, Shihezi, and Altay) from 2015 to 2016. Thus, the uncertainty of corresponding PM_2.5_ satellite-derived values in Xinjiang was evaluated (Figure 2). Linear regression of PM_2.5_ satellite-derived values and ground-based observation values had an acceptable correlation coefficient (R) of 0.830 (*p* ≤ 0.05). And the satellite-derived values were lower than observation values in Xinjiang. As a whole, the satellite-derived PM_2.5_ concentrations were reliable in this study area.

Besides, the driving factor data were mainly obtained from remote sensing data, reanalysis data, and government statistics (Table 1).

The land cover type at yearly intervals (2001–2016) data was provided by MODIS-MCD12Q1 Version 6 data (Land Processes Distributed Active Archive Center, Sioux Falls, United States) [44]. According to the land cover characteristics in Xinjiang and the guide of International Geosphere-Biosphere Programme (IGBP) classification, this study reclassified the land cover types into 8 categories (Forestland, Shrubland, Grassland, Cropland, Urban and Built-Up Land, Snow and Ice, Bare land, Water Bodies). The daytime land surface temperature (LST) derived from MODIS-MCD11A2 were used to identify the spatio-temporal variation of land surface temperature [45]. Vegetation condition was quantified by NDVI provided by MODIS-MOD13Q1 [46]. Additionally, based on black-sky albedo (BSA) and white-sky albedo (WSA) provided by MODIS-MCD43A3 at 500 m spatial resolution and 16-days temporal resolution, we calculate actual albedo, which is interpolated between these two as a function of the fraction of diffuse skylight [47,48]:

(1) $$\alpha_{\mathrm{ac}tual}(\theta_{s})=r(\theta_{s})\alpha_{WSA}+\left[1-r(\theta_{s})\alpha_{BSA}\right],$$

(2) $$r(\theta_{s})=0.122+0.85\times e^{-4.8\mu_{0}},$$

where $\alpha_{\mathrm{ac}tual}$ is the actual albedo; $\alpha_{WSA}$ is the white-sky albedo; $\alpha_{BSA}$ is the black-sky albedo; $\theta_{s}$ is the solar zenith angle which derived from MOD09A1; $\mu_{0}$ is the cosine of the solar zenith angle; $r(\theta_{s})$ is the fraction of diffuse skylight. 

A great deal of research has demonstrated that nightlight data can be used to measure urbanization, economic development, and other socio-economic activities [49,50,51]. Hence, the Defense Meteorological Satellite Program’s Operational Line-Scan System (DMSP-OLS) and the Suomi National Polar-Orbiting Partnership Visible Infrared Imaging Radiometer Suite (NPP-VIIRS) nighttime stable light (NSL) data were used to indicate socio-economic activates in this study. NSL contains the lights from cities, towns, and other sites with persistent lighting, including gas flares. Ephemeral events, such as fires, have been discarded. The National Aeronautics and Space Administration Shuttle Radar Topographic Mission (NASA-SRTM) provided digital elevation model (DEM) data [52]. Based on recent data sets from the Climatic Research Unit (CRU) of the University of East Anglia and the Global Precipitation Climatology Center (GPCC) at the German Weather Service, Köppen-Geiger climate classification maps (2000–2015) were generated [53]. Climate Zone can reflect the regional difference on PM_2.5_ from the prospect of long-term climate characteristics. Two kinds of population (POP) data, which included Asia Continental Population Dataset (2000–2015) and statistical data from the Xinjiang Statistical Yearbook (2017), were used in this research [54]. The socio-economic statistical data included gross domestic product (GDP), and industrial GDP (INGDP) at the county level in Xinjiang was also collected from the Xinjiang Statistical Yearbook (2017). We also downloaded the 2016 OpenStreetMap (OSM) historical dataset from Geofabrik website. OSM road length (Road_L) data and river length (River_L) data in Xinjiang were extracted from the dataset. Based the POP data and areas of counties in Xinjiang, the POP density (POP_D), GDP density (GDP_D), GDP per capita (GDPPC), INGDP density (INGDP_D), INGDP per capita (INGDPPC). Road density (Road_D) and river density (River_D) of each county were calculated. The administrative boundaries of provinces and counties were collected from National Geomatics Centre of China. The acronyms with corresponding full names that are used in this paper were provided in Table 2.

### 2.3. Method

#### 2.3.1. Standard Deviational Ellipse Analysis

The standard deviational ellipse (SDE), which was first proposed by Lefever, can delineate the geographical distribution trend of concerned features [55]. SDE is calculated based on average center of discrete points and the standard distance of other points away from the mean center. Thus, SDE depends on the average location, dispersion, and orientation of spatial points data. The calculated major and minor axes of the ellipse indicate the direction and data distribution range. Based on these theories, SDE is also known as the directional distribution analysis. In this study, the spatial characteristics and the annual moving trace of PM_2.5_ concentrations can reveal the spatial extent, spatial orientation, spatial shape, and spatial center of SDE [15]. As a versatile GIS model for delineating the geographic distribution of spatial points, SDE was calculated by Spatial Statistics Tools in ArcGIS10.6 (ESRI, Redlands, United States).

#### 2.3.2. Spatial Autocorrelation Statistics

Spatial autocorrelation statistics included global spatial autocorrelation and local spatial autocorrelation. Based on the Tobler’s first law of geography, Patrick Moran invented the global Moran’s I which can examine the global spatial autocorrelation patterns of PM_2.5_ concentration and its spatial lag [56]. The Z_I_-score can indicate significant clustering or dispersion of features statistically. Thus, the reliability of Moran’s I (existence of spatial autocorrelation) is tested by using the standardized statistic Z_I_-score. The global Moran’s I and Z_I_-score were calculated by using the below formula:

(3) $$I=\frac{n}{S_{0}}\frac{\sum_{i=1}^{n}\sum_{j=1}^{n}w_{i,j}z_{i}z_{j}}{\sum_{i=1}^{n}z_{i}^{2}},$$

(4) $$Z_{I}=\frac{I-E[I]}{\sqrt{E\left[I^{2}\right]-E{\left[I\right]}^{2}}},$$

where *n* is the number of sample regions; $z_{i}$ is the deviation of an attribute for feature *i* from its mean $\left(x_{i}-\bar{X}\right)$; $\bar{X}$ is the mean of corresponding attribute; $w_{i,j}$ is the spatial weight matrix; $S_{0}$ is the aggregate of all the spatial weights. E[I] is computed as −1/(n−1). The value of global Moran’s I range from −1 to 1. The value less than 0, greater than 0, equal to 0 indicates negative correlation, positive correlation, no correlation, respectively.

The global Moran’s I is the global measurement of the spatial association without identifying the spatial autocorrelation differences among individual region. Therefore, Local Indicators of Spatial Association (LISA) was introduced to interpret the local pockets of nonstationary and location of hot spots [57]. It can also be used to assess the impact of the individual region on global statistics. Here, we use local Moran’s I which is computed as:

(5) $$I_{i}=\frac{x_{i}-\bar{X}}{S_{i}^{2}}\sum_{j=1,j\neq x}^{n}w_{i,j}\left(x_{j}-\bar{X}\right),$$

(6) $$S_{i}^{2}=\frac{\sum_{j=1,j\neq i}^{n}{\left(x_{j}-\bar{X}\right)}^{2}}{n-1}-{\bar{X}}^{2},$$

where *x_i_* is an attribute for feature *I*; $\bar{X}$ and $w_{i,j}$ are the same as in Equation (3).

The Z_I_-score was also used to indicate the significance level of the LISA model. Generally, the LISA map, which consists of four types of spatial autocorrelation (“High-High”, “Low-Low”, “High-Low”, “Low-High”), was divided by local Moran’s I, Z_I_-score and *p* value of local Moran’s I. Regions whose Z_I_-scores were not statistically significant at the 5% level (*p* ≤ 0.5) will show as “Not Significant”. The relationships between them are as follows (Table 3).

The ArcGIS10.6 and GeoDa1.12 software was implemented to calculate the global and local Moran’s I in this research.

#### 2.3.3. Geographical Detector Method

Based on the spatially stratified heterogeneity, which refers to the phenomena that within strata are more similar than between strata, the fundamental theory of the geographical detector method was first proposed by Wang in 2010 [23]. The geographical detector method applies *q* value to quantitatively measure the heterogeneity and autocorrelation of the dependent variable, and detects the association between the dependent variable and its influencing factors. In this research, GDM was used to assess the non-linear associations between PM_2.5_ concentration and its natural and social-economic factors. The *q* value of GDM was calculated as follows:

(7) $$q=1-\frac{\sum_{h=1}^{L}N_{h}\sigma_{h}^{2}}{N\sigma^{2}},$$

(8) $$\sigma^{2}=\frac{1}{N}\sum_{i=1}^{N}(R_{i}-\bar{R}{)}^{2},$$

(9) $$\sigma_{h}^{2}=\frac{1}{N}{\sum_{h=1}^{L}\sum_{j=1}^{N_{h}}\left(R_{h,j}-{\bar{R}}_{h}\right)}^{2},$$

where *N* refers to the total number of samples in the entire study area, and $\sigma^{2}$ represents the global variance of response variable Y in the entire study area. In this study, Y means the PM_2.5_ concentration. The study area was stratified into *L* zones (*h* = 1, …, *L*), and the stratification depends on the characteristics of the explanatory variables (X). In the study, X means the driving factor, such as LCT, albedo, NDVI. $N_{h}$ and $\sigma_{h}^{2}$ represent the number of samples and the stratified variance of Y within *h*-th zone, respectively. $R_{i}$ and $R_{h,j}$ refer to the value of the *i*-th and *j*-th samples from the whole study area and *h*-th zone, respectively. $\bar{R}$ and ${\bar{R}}_{h}$ stand for the mean value of samples in all the regions and *h*-th zone, respectively.

From Equation (5), we can see that the *q* value lies between 0 and 1. It means that the *q* value is 1 only when X completely determines Y. Otherwise, if X is completely unrelated to Y, the *q* value will be 0.

According to the introduction of GDM [58], the model consists of the following four modules:

(1) The factor detector calculates the determinant power of an explanatory variable X of Y, which is the *q* value we mentioned above.
(2) The risk detector maps the average value of response variable in each stratum (zone). It can be used to compare the difference of average PM_2.5_ concentration values between sub-regions.
(3) The interaction detector can reveal the interactive effect of X1 and X2 on Y. In other words, that is the relationship among *q*(X1), *q*(X2), and *q*(X1∩X2).
(4) The ecological detector identifies the statistic difference of the impacts between X1 and X2. It can show the relative importance between these two factors.

By means of the relationship among *q*(*X1*), *q*(*X2*), and *q*(*X1*∩*X2*), the interactive effect was catalogued as the following Table 4.

#### 2.3.4. Technical Flowchart of This Study

**Figure 3:**
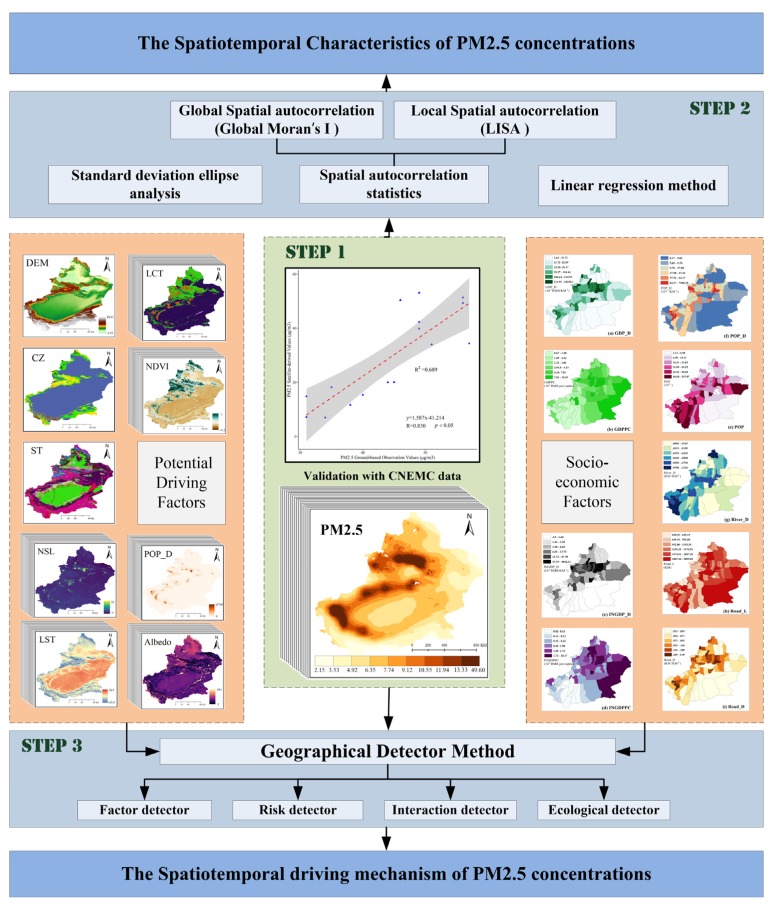
The technical flowchart of this study.

Based on the objective of this study, this manuscript is organized as presented in the technical flowchart (Figure 3). The research consists of three main steps: First, we validated the accuracy of annual PM_2.5_ concentrations grid data with ground-based observation values downloaded from the CNEMC website. Second, based on the linear regression method, SDE analysis and spatial autocorrelation statistics, we explored the spatio-temporal characteristics of PM_2.5_ concentrations. Finally, we applied geographic detector method to quantitatively evaluate the effects of socio-economic factors on PM_2.5_ concentrations in 2016. Moreover, interannual variation of other potential driving factors for PM_2.5_ concentration was explored during 2001–2016.

## 3. Results

### 3.1. The Spatio-Temporal Characteristics of PM_2.5_ Concentrations

#### 3.1.1. The Spatio-Temporal Pattern and Variation of PM_2.5_ Concentrations

**Figure 4:**
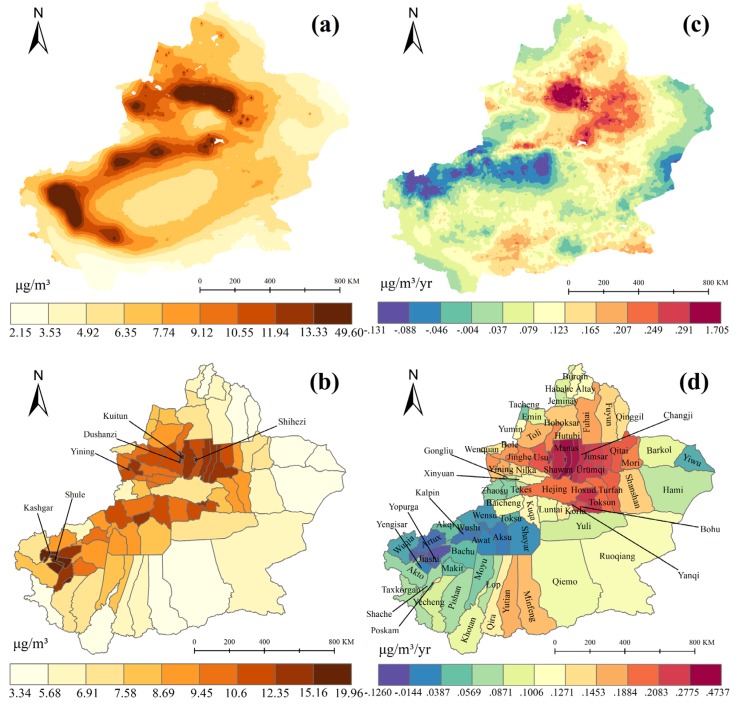
Spatial distributions of (**a**,**b**) average annual PM_2.5_ concentrations (μg/m³), (**c**,**d**) linear trends of annual PM_2.5_ concentrations (μg/m³/yr) in Xinjiang from 2001 to 2016. Figure 4b,d represents the average annual PM_2.5_ concentrations (μg/m³) and linear trends of annual PM_2.5_ concentrations (μg/m³/yr) at the county level.

Based on annual average satellite-based PM_2.5_ concentration data (2001–2016) and the Xinjiang political districts map, average annual PM_2.5_ concentration in the whole region (a) and different county (c) were calculated. As Figure 4a,c shows, there exists a significant spatial difference of PM_2.5_ concentration exited in Xinjiang. PM_2.5_ concentrations were higher in urban agglomeration located in the northern Tianshan Mountain and western Tarim Basin, especially in Shihezi (19.96 μg/m^3^), Kashgar (19.67 μg/m^3^), Shule (18.09 μg/m^3^), Yining (17.51 μg/m^3^), Kuitun (17.42 μg/m^3^), Dushanzi (16.50 μg/m^3^). The PM_2.5_ concentration in 33 cities or counties exceeds the 10 μg/m^3^ which is the WHO average annual limit of primary PM_2.5_ standards. However, PM_2.5_ concentrations were lower in the sparsely-populated area in eastern and southern Xinjiang. Furthermore, based on linear regression analysis, PM_2.5_ concentration interannual trends were calculated. Figure 4b,d shows a finding in northern Tianshan, where PM_2.5_ concentrations were increased at an annual rate of 1.1–1.7 μg/m^3^/yr. PM_2.5_ concentrations in several counties surrounded by Urumqi and Changji were increased significantly from 2001–2016. While in the western Tarim Basin, PM_2.5_ concentrations were decreased with the rates ranging from −0.1–0.07 μg/m^3^/yr, especially in Kashgar.

**Figure 5:**
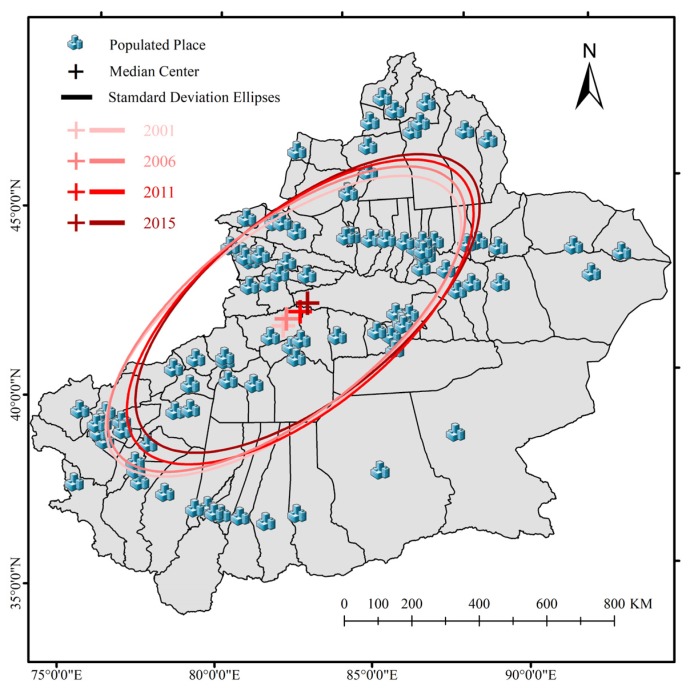
Spatial changes of the median center and standard deviation ellipses of PM_2.5_ concentrations.

Based on SDE analysis and the PM_2.5_ concentrations in 106 populated places of Xinjiang (more than 200 persons per square kilometers), Figure 5 shows that the main distribution of PM_2.5_ concentrations was aligned in the southwest–northeast direction. Additionally, the median center made a clear but gradual move from southwest to northeast. This movement mainly caused by the rapid increase of the high PM_2.5_ concentrations in the northern slope of the Tianshan Mountain. Moreover, the area of standard deviational ellipse increased at first and then decreased during the study period. The spatial distribution of PM_2.5_ concentrations presented a trend of gradual concentration after dispersion from 2005. Additionally, the ratios of standard deviational ellipse principal and auxiliary axis lengths showed the decrease from 2001 to 2011 and the increase from 2011–2015. The accumulation degree of PM_2.5_ concentrations associated with the northwest–southeast direction was higher from 2001–2011, whereas those associated with the northeast–southwest direction became higher in 2011. 

#### 3.1.2. The Spatial Agglomeration Law of PM_2.5_ Concentrations

**Figure 6:**
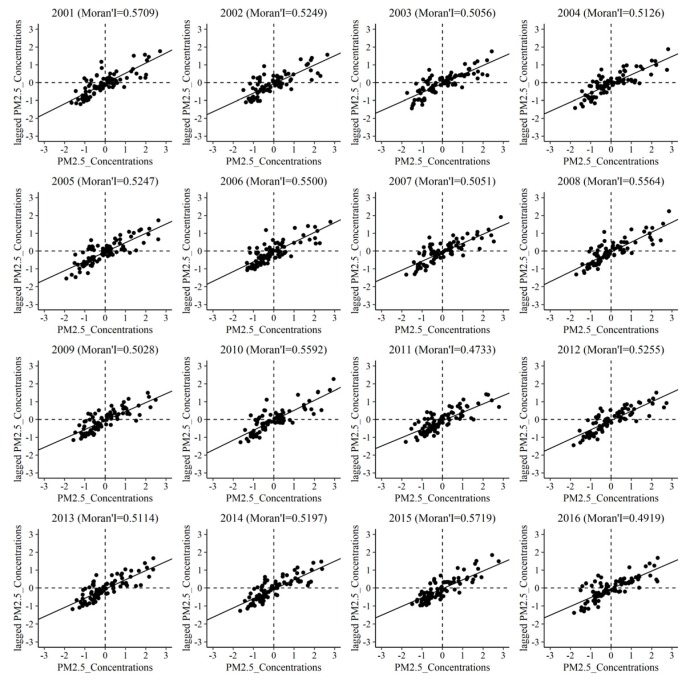
Global Moran’s I scatterplots of PM_2.5_ concentrations (2001–2016).

**Figure 7:**
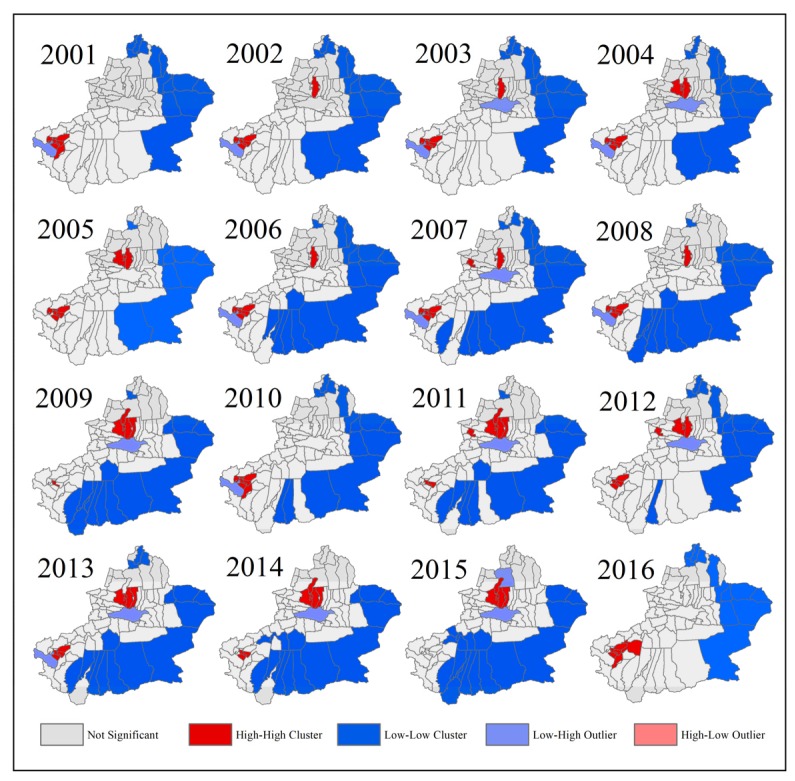
Spatial agglomeration diagram (LISA map) of PM_2.5_ concentrations (2001–2016).

Spatial autocorrelation analysis, including the global Moran’s I scatter plot and LISA agglomeration analysis, was used to quantitatively analyze the spatial agglomeration laws of PM_2.5_ concentrations from 2001 to 2016. Figure 6 shows the global Moran’s I scatter plots of PM_2.5_ concentrations in Xinjiang from 2001 to 2016. In the scatterplots, the horizontal axis represents the standardized PM_2.5_ concentration in each country, and the vertical axis represents the neighboring PM_2.5_ concentration value calculated by the spatial weight matrix based on the Euclidean distance, also called lagged PM_2.5_ concentrations. It is worth noting that the Moran’s I values show a trend of decline, with maximum value of 0.5733 and minimum of 0.4719, which are all positive and significant (*p* ≤ 0.01) within the study period. Most of the dots concentrated in the first and third quadrants, meaning that most counties show the positive spatial autocorrelations of PM_2.5_ concentrations. This can be explained by the High-High cluster and Low-Low cluster in the LISA map (Figure 7). Similarity, the country which showed the Low-High cluster and High-Low cluster should appear in the second and fourth quadrants. As shown in Figure 7, there exists a slight increase among counties which showed as the High-High cluster from 2001 to 2015. Ruoqiang, Hami, Yiwu, and Barkol manifest as the Low-Low cluster every year, whereas no counties manifest as the High-High cluster in each year. Overall, High-High clusters mainly distributed in the northern slope of the Tianshan Mountain and western Tarim Basin, while Low-Low clusters mainly distributed in southern and eastern Xinjiang.

### 3.2. The Effect of Socio-Economic Factors on PM_2.5_ Concentations

**Figure 8:**
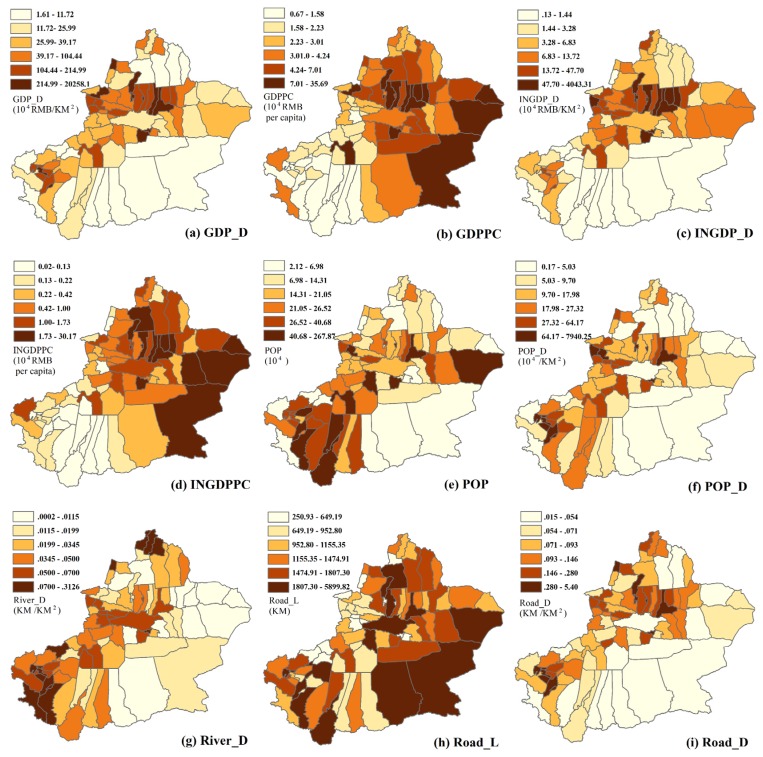
Spatial distributions of socio-economic factors. (**a**) GDP density (GDP_D), (**b**) GDP per capita (GDPPC), (**c**) industrial GDP density (INGDP_D), (**d**) industrial GDP per capita (INGDPPC), (**e**) population (POP), (**f**) population density (POP_D), (**g**) river density (River_D), (**h**) road length (Road_L), (**i**) road density (Road_D). All factors are discretized from continuous variables to categorical variables by quantile method.

**Figure 9:**
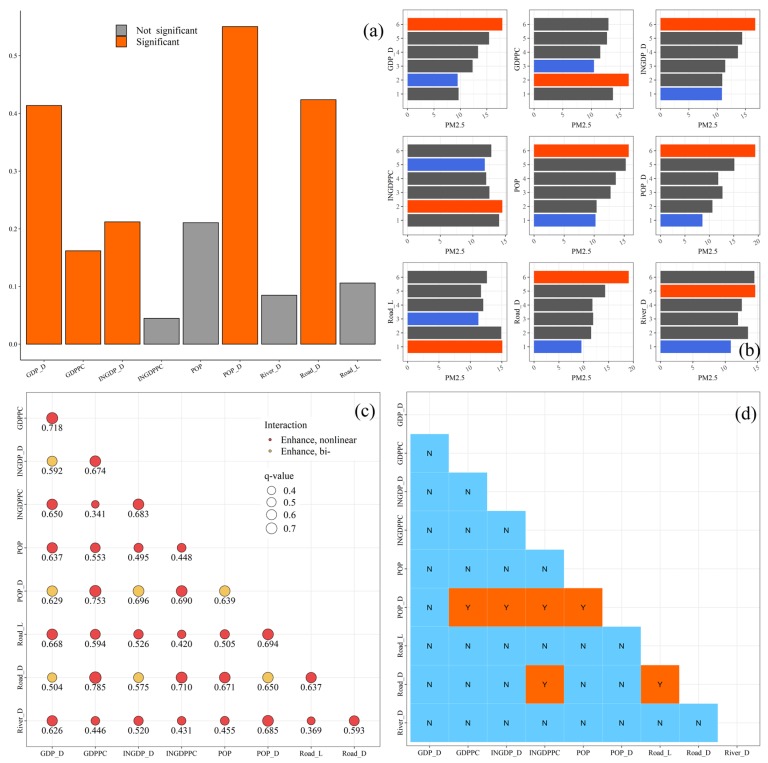
The result of GDM: (**a**) factor detector, (**b**) risk detector, (**c**) interaction detector, (**d**) ecological detector. Note: In Figure 9b, the orange bar means the maximum value in the sub-histogram and the blue bar means the minimum value in the sub-histogram.

Due to the input variables of GDM that must be the categorial variable, here we used the quantile method as the discretization method to transform the numerical variables into categorial variables (Figure 8). The dependent variable are as follows, GDP_D, GDP, GDPPC, INGDP_D, INGDPPC, POP, POP_D, Road_L, Road_D, River_D. As shown in Figure 9a, the factor detector showed that population density was the dominant factor on the spatial distribution of PM_2.5_ concentrations (*q* = 0.550), followed by road network density (*q* = 0.423), GDP density (*q* = 0.413), INGDP density (*q* = 0.212), GDP per capita (*q* = 0.161). The results of other factors were not significant at the *p* ≤ 0.05 level. According to the risk detector module of GDM, the average PM_2.5_ concentrations in each stratum of different factors was calculated (Figure 9b). The counties with higher GDP_D, INGDP_D, Road_D, POP, and POP_D have more serious air pollution problem. This illustrates that there exists a positive correlation between these factors and PM_2.5_ concentrations. As shown in Figure 9c, the interaction between any two factors can enhance their explanatory power for the spatial distribution in PM_2.5_ concentrations. The dominant interactions between GDPPC and Road_D show the highest *q* values (*q* = 0.785), followed by GDPPC∩POP_D (*q* = 0.753), GDPPC∩GDP_D (*q* = 0.718), and INGDPPC∩Road_D (*q* = 0.710). In addition, these interactions all belonged to the bivariate enhancement interaction (*q*(X1∩X2) > *q*(X1) + *q*(X2)). Although GDPPC was not the strongest explanatory power for the spatial pattern of PM_2.5_ concentrations, the interactive explanatory power between GDPPC and other socio-economic factors were surprisingly high. Additionally, the ecological detector result shows that the POP_D has a significantly stronger effect on PM_2.5_ than other socio-economic factors, except GDP_D (Figure 9d).

### 3.3. Interannual Variation of Potential Driving Factors for PM_2.5_ Concentrations 

As described in the Section 3.1, PM_2.5_ concentrations gradually changed in terms of spatio-temporal distribution. In addition, the spatial aggregation pattern of regional PM_2.5_ concentrations was easily affected by socio-economic and natural factors. In order to identify the determinant power and its interannual variation of potential driving factors more comprehensively, the total 9 potential driving factors of PM_2.5_ concentrations, including LCT, CZ, Albedo, POP_D, NSL, LST, NDVI, and DEM, were selected. The q-value in GDM was used to describe the interannual variation of PM_2.5_ potential driving factors during 2001–2016.

**Figure 10:**
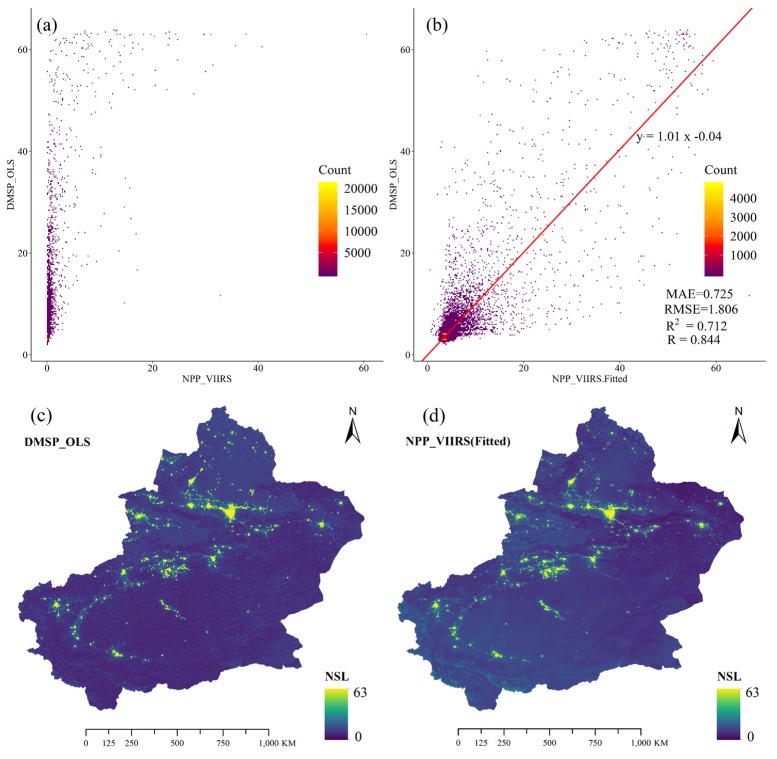
The relationship between DMSP_OLS NSL and before and after NPP_VIIRS NSL fitted in 2013 (**a**,**b**), and the spatial distribution of DMSP_OLS NSL (**c**) and NPP_VIIRS NSL fitted in 2013 (**d**).

**Figure 11:**
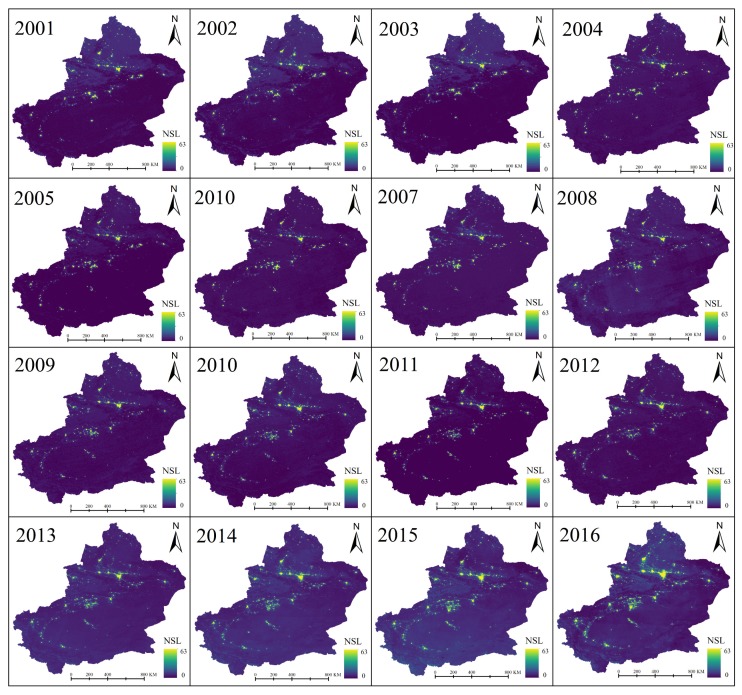
Spatial distributions of annual average NSL in Xinjiang from 2001–2016.

Due to the absence of spatial continuous and reliable long time series of socio-economic data, nighttime stable light (NSL) data with high spatial resolution data were used to provide a proxy to the infrastructure and economic development in this study area. However, National Geophysical Data Center (NGDC) stopped producing monthly composites of DMSP_OLS data after February 2013, while NPP/VIIRS data, which has supplied from April 2012, is a follow-up to DMSP_OLS data. In this study, an exponential model was used to fit the two data sources which were desaturated and resampled to 1 km. The mean absolute error (MAE), root mean square error (RMSE), determination coefficient (R^2^), and the Pearson correlation coefficient R between two data sources were calculated to evaluate model fitting effects (Figure 10b), and a good fit was shown (R^2^ = 0.712). The more intuitive NSL fitting results are shown in Figure 10cd. Based on the exponential model, long-term (2001–2016) annual average NSL data were generated as shown in Figure 11. The NSL in all major cities of Xinjiang is gradually brightening and the NSL coverage area is gradually increasing. The expansion of city size was easy to identify in Xinjiang, especially in NTMEZ. But in the western Tarim Basin, the growth trend of city size was less obvious. 

**Figure 12:**
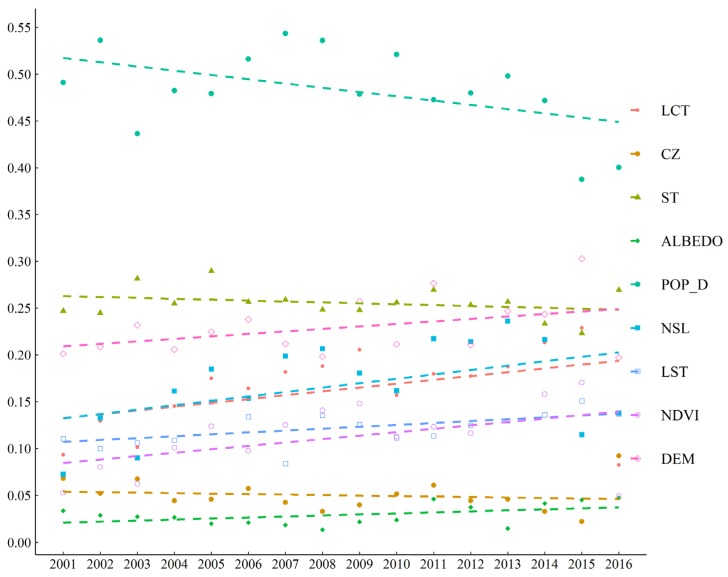
Trend analysis of *q* value of each driving factors during 2001–2016 for Xinjiang.

Based on the continuous grid data, we explored the variations of driving forces on PM_2.5_ spatial distribution by using GDM from 2001–2016. The driving factors are mainly divided into two parts: social-economic factors and natural factors, both of which can affect the formation, distribution maintenance, and change of PM_2.5_ concentrations [21]. All the driving factors passed the significance test at a significance level of 0.01 (*p* ≤ 0.01). As shown in Figure 12, the driving factors are sorted by average *q* values during the study period (2001–2016) as follows: POP_D (0.483) > ST (0.256) > DEM (0.229) > NSL (0.167) > LCT (0.122) > NDVI (0.112) > CZ (0.050) > Albedo (0.029). Although the POP_D was still the most important driving factor, the explanatory power decreased significantly, especially from 2007. Conversely, DEM, NSL, LCT, and NDVI showed the increased trend on the driving forces of PM_2.5_ concentrations during the study period. The *q* value of NSL showed the most rapid increase of all the driving factors. Moreover, the *q* value of ST, CZ and Albedo showed stable and vibration path.

## 4. Discussion

Given the paucity of comprehensive studies about the spatio-temporal variations in PM_2.5_ concentrations in the whole Xinjiang, we have systematically analyzed spatio-temporal characteristics of PM_2.5_ concentrations and its natural social economy determinants. First of all, an overall agreement was estimated between satellite-based and ground observed PM_2.5_ concentration data in Xinjiang, with an acceptable correlation coefficient of 0.830. This result reveals the fitness of satellite-based PM_2.5_ concentration data, especially in north Xinjiang. However, due to the short set-up time of the current ground PM_2.5_ monitoring station, we only used the data from the two complete ground observation years of 2015 and 2016 to compare with the satellite-based PM_2.5_ concentration data.

From the analysis results of this study, we can see that the north slope of the Tianshan Mountain and the western Tarim Basin are the major source areas of PM_2.5_ in Xinjiang. Coincidentally, these two areas are the most densely populated places, as well as most economically prosperous regions in Xinjiang [59,60]. From 2001 to 2015, the mean center of PM_2.5_ concentrations in Xinjiang showed a notable move to the northeast by reason of the rise of PM_2.5_ concentrations in the north slope of the Tianshan Mountain. In the north slope of the Tianshan Mountain, with the increasing numbers of backward industries, such as labor-intensive and energy-intensive industries, the PM_2.5_ concentrations went obviously higher in recent years. The Zhundong Coalfield is China’s largest intact coalfield, located in Changji (Figure 1). Exploited since 2006, the Zhundong Coalfield has become one of the main thermal power and coal chemical industrial bases of Xinjiang [61]. The gradual concentration trend of PM_2.5_ concentrations in the north slope of the Tianshan Mountain from 2005 indicated some environment effects of Zhundong Coalfield mining activities. With collecting and analyzing daily PM_2.5_ samples in Dushanzi, Tura, et al. [30] found that coal combustion and soil dust contributions accounts for nearly half of PM_2.5_ sources. A great deal of research shows that the coal mining operations, coal transport, coal processing, and coal burning would all generate significant atmospheric pollution [62,63]. Therefore, we have sufficient reason to believe that the increase oin heavy energy consumption enterprises and mining enterprises are one of the main reasons for the increase in PM_2.5_ concentrations on the northern slope of Tianshan. Although these industries are the main source of local tax income and the main provider of employment, the government should focus on rectifying exhaust emissions from these factories. Environmental protection supervision and punishment of mining companies should be increased, especially for dust-prone industries such as open-pit coal mines. While controlling pollution at the source, the Ecological Environment Bureau should strengthen supervision during coal transportation, storage, and processing. Most importantly, government should accelerate wind and solar power development, which can reduce the proportion of coal in electricity generation. Meanwhile, the construction of eco-industrial parks should be promoted in Xinjiang, which could be an effective way to realize green economic growth and sustainable development [31]. 

In order to further explore the driving factors of PM_2.5_ concentrations, we used the geographic detector method (GDM) to quantify the interannual change of driving forces. The results show that population was still the greatest power of determinant (*q* = 0.550) on the spatial distribution of PM_2.5_ concentrations in Xinjiang. This is basically consistent with the results of previous studies on attribution of PM_2.5_ in eastern China or throughout China [21,64,65,66]. This suggests that PM_2.5_ mainly comes from human activities, which includes more motor vehicles and coal burned for heating in winter. Due to rapid urbanization and development of heavy industry, the contribution of the single factor like population density has become less influential in Xinjiang. Therefore, as shown in Figure 12, the impact of population density on PM_2.5_ concentrations shows a falling trend since 2008. Even so, population is still the most important driving factor affecting the spatial distribution of PM_2.5_ concentrations. Moreover, GDP per capita and road network density show the strongest interaction (*q* = 0.785) on the explanatory power of the spatial distribution of PM_2.5_ concentration. From the perspective of social and economic development, the combination of GDP and road network density can represent economic activity to a certain degree. Consequently, Xinjiang’s environment has been sacrificed to achieve rapid economic growth in recent years, especially the air quality. Considering that nighttime stable light (NSL) can describe social and economic activity more effectively, long time series NSL data was reconstructed as one of driving factors for PM_2.5_ concentration distribution [13]. Liu et al. [67] found the effectiveness and potential values of using NSL, NDVI, and elevation on improving the accuracy and spatial resolution of the satellite-based PM_2.5_ concentration dataset. 

Indeed, as shown in Figure 12, some factors, like NSL and LCT, which can represent the city size and the level of economic activity, play a more and more important role along the study period. In the past nearly twenty years, the social and economic situation of Xinjiang has undergone tremendous changes. According to the China Statistical Yearbooks in 2001 and 2019, Xinjiang’s private car ownership has increased from 0.135 million to 3.2921 million, and the annual electricity consumption increased from 18.3 billion kWh to 213.8 billion kWh [41,68]. Hence these findings suggest that the rapid expansion of the urban area should slow down and new energy vehicles should be promoted in the future, especially in NTMEZ. Furthermore, the explanatory power of the natural factor, like DEM NDVI, also plays an increasingly important role in controlling the formation of PM_2.5_ (Figure 12). X, et al. [69] found that the diversity of the complex landforms of Xinjiang is the main cause of the spatial complexity of the precipitation distribution. It is generally accepted that rainfall has a significant inhibitory effect on the formation of PM_2.5_. On the other hand, due to the popularity of agricultural mechanized production and the overuse of water resources, a large amount of bare land has been converted into cropland in Xinjiang [70,71]. By analyzing the MODIS land cover data, we conclude that the cropland area was about 4.76 × 10^4^ km^2^ in 2001, while in 2016, the cropland area increased to 7.18 × 10^4^ km^2^. In addition, the bare land area has been reduced more than 3.49 × 10^4^ km^2^ in Xinjiang. Based on the GIMMS NDVI3g and MODIS NDVI data, there exists an increasing trend of vegetation greenness in Xinjiang from 2001 to 2015 [42,72]. The regions with higher vegetation coverage and greenness have the strong removal and absorption capacity for PM_2.5_. With the increase in the artificial oasis area along the edge of the Tarim desert for the last 16 years, the ecological environment of the desert edge cities has improved, and the absorption capacity of farmland on PM_2.5_ has also increased. Considering that vegetation can accelerate the settlement rate of PM_2.5_, under the premise of effective use of limited water resources, the area of urban green space should be increased through afforestation in barren mountains. Green land can reduce the PM_2.5_ concentrations of cities while preventing the spread of sand and dust in the suburbs and deserts. 

## 5. Conclusions

Based on standard deviation ellipse analysis and spatial autocorrelation statistics, this study analyzed the spatio-temporal variation of satellite-based PM_2.5_ concentrations in Xinjiang from 2001 to 2016. We investigated the socio-economic and natural factors affecting PM_2.5_ concentration by using the geographic detector method (GDM). Based on our findings, the main conclusions are as follows. From 2001 to 2016, almost 40% of cities or counties in Xinjiang exceeded the WHO’s annual standard value of 10 μg/m3. The PM_2.5_ concentration was higher in the northern slope of the Tianshan Mountains and the western Tarim Basin. Due to the rapid development of the North Tianshan Mountain Economic Zone (NTMEZ) in the past ten years, the PM_2.5_ concentration has gradually increased along the northern slopes of the Tianshan Mountains, while it has decreased significantly in the western Tarim Basin. Population density is the dominant factor affecting the spatio-temporal variation of PM_2.5_ concentration, followed by road network density and GDP density. The city size and its economic development will increasingly affect the spatial distribution of PM_2.5_ concentrations in the future, while the effect of population density will gradually decrease. Another interesting finding is that the complex landforms and vegetation conditions have a potential relationship with the spatio-temporal variation of PM_2.5_ concentration in Xinjiang. This is an interesting topic for future work, and more detailed research should be conducted.

This study, for the first time, reveals spatio-temporal variability and its attribution of PM_2.5_ concentration in northwest China. The results of the study will help the local government to formulate new and effective environmental policies.

## Figures and Tables

**Table 1:** Summary information on the used datasets.

Dataset	Data Sources	Spatial Resolution	Temporal Resolution
LCT	MODIS MCD12Q1 (2001–2016)	500 m	1 year
LST	MODIS MOD11A2 (2001–2016)	1000 m	8 day
NDVI	MODIS MOD13Q1 (2001–2016)	250 m	16 day
Albedo	MODIS MCD43A3 (2001–2016)	500 m	1 day
NSL	DMSP-OLS (2001–2013) /NPP-VIIRS (2013–2016)	1000 m/500 m	1 year/ 1 month
DEM	NASA Shuttle Radar Topographic Mission	90 m	-
CZ	Köppen-Geiger climate classification maps (2000–2015)	1 km	-
POP	Asia Continental Population Dataset (2000–2015) and 2017 Xinjiang Statistical Year book	1 km	5 year
GDP	2017 Xinjiang Statistical Year book	-	-
INGDP	2017 Xinjiang Statistical Year book	-	-
Road_L	OpenStreetMap historical dataset	-	-
River_L	OpenStreetMap historical dataset	-	-

**Table 2:** Complete list of acronyms with corresponding full names used in this paper.

Acronyms	Full Name	Acronyms	Full Name
ACAG	Atmospheric Composition Analysis Group	LST	Land surface temperature
AERONET	Aerosol Robotic Network	MAE	Mean Absolute Error
AOD	Aerosol Optical Depth	MAIAC	Multi-angle implementation of atmospheric correction
BSA	Black-sky albedo	MISR	Multiangle Imaging Spectroradiometer
CNEMC	Chinese National Environmental Monitoring Center	LISA	Local Indicators of Spatial Association
CRU	Climatic Research Unit	MODIS	Moderate Resolution Imaging Spectroradiometer
CZ	Climate Zone	NASA-SRTM	National Aeronautics and Space Administration Shuttle Radar Topographic Mission
DB	Deep Blue	NDVI	Normalized Difference Vegetation Index
DEM	Digital elevation model	NGDC	National Geophysical Data Center
DMSP-OLS	Defense Meteorological Satellite Program’s Operational Line-Scan System	NPP-VIIRS	Suomi National Polar-Orbiting Partnership Visible Infrared Imaging Radiometer Suite
DT	Dark Target	NSL	Nighttime stable light
GBD	Global Burden of Disease	NTMEZ	North Tianshan Mountain Economic Zone
GDM	Geographical detector method	OSM	OpenStreetMap
GDP	Gross Domestic Product	PFCs	Per fluorinated compounds
GDP_D	GDP density	PM_2.5_	Particulate matter with a diameter of 2.5 μm or less
GDPPC	GDP per capita	POP	Population
GIMMS	Global Inventory Modelling and Mapping Studies	POP_D	POP density
GPCC	Global Precipitation Climatology Center	RMSE	Root Mean Square Error
GTWR	Geographically and Temporally Weighted Regression	Road_L	Road length
GWR	Geographically Weighted Regression	Road_D	Road density
HMs	Heavy metals	River_L	River length
IGBP	International Geosphere-Biosphere Programme	River_D	River density
INGDP	Industrial GDP	SDE	Standard deviational ellipse
INGDP_D	INGDP density	SeaWiFS	Sea-Viewing Wide Field-of-View Sensor
INGDPPC	INGDP per capita	WHO	World Health Organization
LCT	Land cover type	WSA	White-sky albedo

**Table 3:** Spatial autocorrelation types of LISA map.

Local Moran’s I	*p* Value	Z_I_-Score	Spatial Autocorrelation Types
Positive	*p* > 0.05	Z_I_ > 0	High-High Cluster
Positive	*p* > 0.05	Z_I_ < 0	Low-Low Cluster
Negative	*p* > 0.05	Z_I_ > 0	High-Low Outlier
Negative	*p* > 0.05	Z_I_ < 0	Low-High Outlier

**Table 4:** Types of interaction relationships between two factors.

Interaction Type	Description
Weaken, univariate	Min(*q*(*X1*), *q*(*X2*)) < *q*(*X1*∩*X2*) < Max(*q*(*X1*)), *q*(*X2*))
Weaken, non-linear	*q*(*X1*∩*X2*) < Min(*q*(*X1*), *q*(*X2*))
Enhance, bivariate	*q*(*X1*∩*X2*)> Max(*q*(*X1*), *q*(*X2*))
Enhance, non-linear	*q*(*X1*∩*X2*) > *q*(*X1*) + *q*(*X2*)
Independent	*q*(*X1*∩*X2*) = *q*(*X1*) + *q*(*X2*)
